# The Hidden Cost of Untreated Paragangliomas of the Head and Neck: Systemic Reactive (AA) Amyloidosis

**DOI:** 10.1155/2015/250604

**Published:** 2015-03-09

**Authors:** Erkan Dervisoglu, Murat Ozturk, Mehmet Tuncay, Gulhatun Kilic Dervisoglu, Yesim Gurbuz, Serhan Derin

**Affiliations:** ^1^Department of Nephrology, School of Medicine, Kocaeli University, 41000 Kocaeli, Turkey; ^2^Department of Otorhinolaryngology, School of Medicine, Kocaeli University, 41000 Kocaeli, Turkey; ^3^Department of Neurology, Kandira Government Hospital, 41600 Kocaeli, Turkey; ^4^Department of Pathology, School of Medicine, Kocaeli University, 41000 Kocaeli, Turkey; ^5^Department of Otorhinolaryngology School of Medicine, Muğla Sıtkı Koçman University, 48000 Muğla, Turkey

## Abstract

We report a case of a 51-year-old man who was diagnosed with systemic reactive (AA) amyloidosis in association with untreated glomus jugulare and glomus caroticum tumors. He refused radiotherapy and renal replacement therapy. Paragangliomas, although rare, should be considered one of the tumors that can result in AA amyloidosis.

## 1. Introduction

Paragangliomas are uncommon true neoplasms that arise from paraganglionic chemoreceptor cells. Other terms used to describe these lesions include glomus tumors, nonchromaffin paragangliomas, chemodectomas, and carotid body-like tumors [1]. Systemic reactive (AA) amyloidosis is usually observed during chronic infectious or inflammatory processes or rarely with benign or malignant tumors. The association of isolated paragangliomas with systemic reactive amyloidosis has been rarely reported. In this report, we present a case of systemic reactive amyloidosis associated with glomus jugulare and caroticum tumors.

## 2. Case Report

A 51-year-old man with a glomus jugulare tumor was referred to our nephrology clinic with suspected renal failure and proteinuria. Twenty-nine years prior, the patient had an operation for glomus jugulare tumor that resulted in extensive hemorrhaging and required a two-month hospitalization. Since the operation, the patient had not sought any follow-up care for his glomus tumor. His brother was diagnosed as having a glomus caroticum tumor. The patient complained of pulsatile tinnitus and a mass in his right external ear canal.

**Figure 1:**
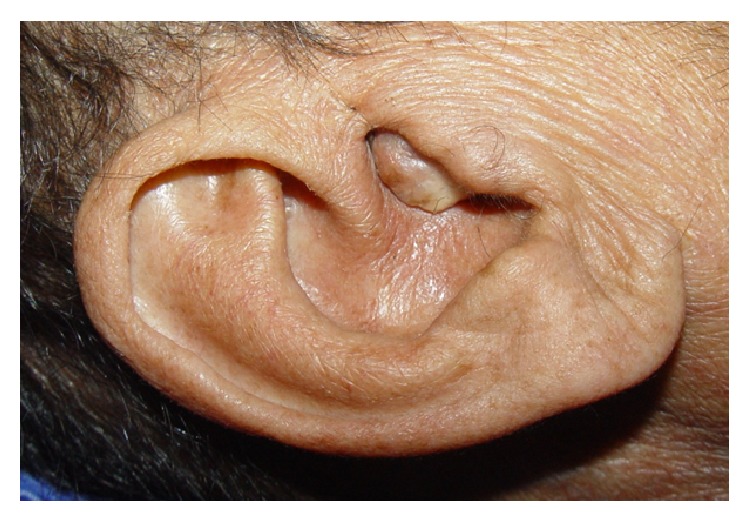
A red-whitish mass protruding from the middle ear to external ear, filling the external ear canal.

**Figure 2:**
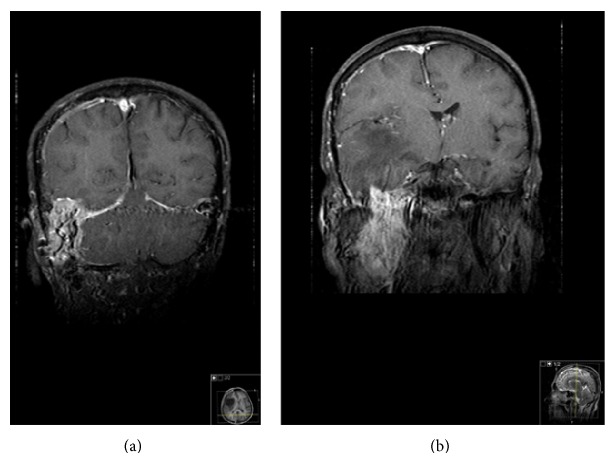
Cranial magnetic resonance images demonstrate (a) a mass lesion that arises from the right jugular foramen, extending to the medial cranial fossa, consistent with glomus jugulare, and (b) a mass lesion at the carotid bifurcation, consistent with glomus caroticum.

On admission, the patient's blood pressure was 100/70 mm Hg, pulse rate 78/min, and temperature 36.6°C. He had 1+ pitting edema in the lower extremities. On otolaryngologic exam, the patient had a red-whitish mass protruding from the middle ear to external ear, filling the external ear canal (Figure 1). Audiometry testing found an 85 dB mixed-type hearing loss on the left and a 29 dB sensorineural hearing loss on the right. The patient had a daily and urinary protein excretion of 2 g and albumin excretion of 1 g with no abnormality in urinary sedimentation. Erythrocyte sedimentation rate was 69 mm/h and C-reactive protein level was 6.4 mg/dL (normal: <0.8 mg/dL). His hemoglobin was low (9.33 g/dL), but his platelet and leukocyte counts were normal. Serum creatinine was 4.09 mg/dL, blood urea nitrogen 46 mg/dL, total protein 5.9 g/dL, and albumin 2.86 g/dL. Serum electrolytes, liver enzymes, and C3 and C4 levels were within normal range. Tests for hepatitides B and C, HIV, anti-nuclear antibody, and anti-double-stranded DNA were also negative. Chest X-ray and EKG were normal. Abdominal ultrasonography showed normal sized kidneys with bilateral grade 2 echogenicity. After placement of a dual-lumen hemodialysis (HD) catheter into the right internal jugular vein, HD treatment three times weekly was started. Cranial magnetic resonance imaging (MRI) showed a mass lesion arising from the right jugular foramen, extending to medial cranial fossa (consistent with a glomus jugulare), and a mass lesion at the carotid bifurcation, consistent with a glomus caroticum (Figure 2). The tumors were deemed unresectable and radiotherapy was suggested.

**Figure 3:**
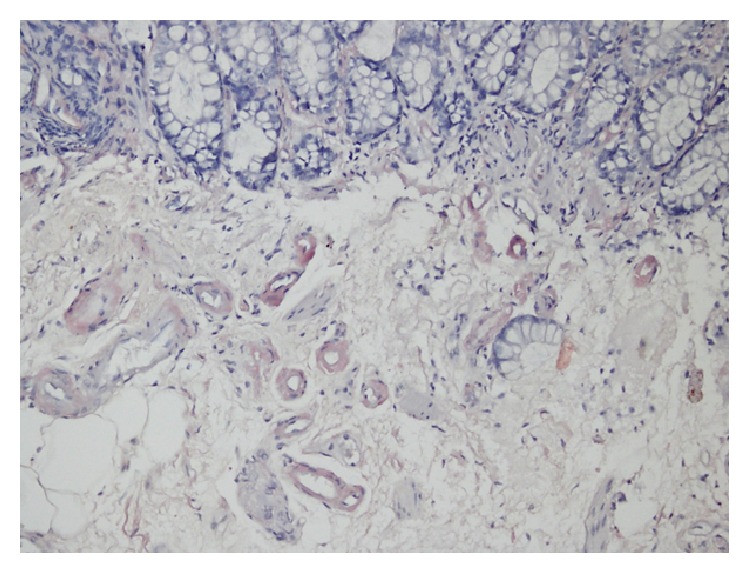
Amyloid is noted in the submucosal vessels (Congo red stain ×100).

We suspected amyloidosis as the underlying cause of his renal failure and proteinuria. Due to his severe renal failure, we performed a rectal biopsy without complication. A homogenous, amorphous, and eosinophilic material was seen in the walls of submucosal vessels on light microscopy. The material was positive on Congo red staining and was digested by KMnO_4_ (Figure 3). Immunohistochemical analysis showed positive staining for AA amyloid. Based on these results, we made a diagnosis of AA amyloidosis. Neither he nor his family had a history of Mediterranean fever (MEFN), and he had no clinical signs of MEFN. Treatment was started with colchicine 1 mg/day and the patient continued with regular HD three times weekly. He refused to receive radiotherapy for his tumors and died 3 months after the commencement of HD.

## 3. Discussion

Paraganglionic tissue is derived from the neural crest and is found in several locations in the body: in the carotid body, along the course of Jacobson's nerve, within the adventitia of the jugular bulb at the jugular foramen, and within the adrenal medulla [2]. Jugular foramen paragangliomas are deep-seated, extremely vascular lesions that involve vital neurovascular skull base structures. First line treatment is total surgical excision of the tumor. If excision is not possible because of the particular anatomy or because of factors associated with a high risk of surgical complications, radiation therapy may be used. Irradiation induces a vasculitis, which halts tumor progression [3]. In the present case, because clinical and radiographic findings revealed tumor spread to several different locations, we offered radiotherapy as treatment, but the patient refused.

The association of isolated paragangliomas with systemic reactive amyloidosis has been rarely reported. Amongst paragangliomas associated amyloidosis there were two reports of glomus caroticum tumors [4, 5]. In one of them tumor was isolated unilaterally [4], and, in the other, tumor was a part of familial multiple endocrine neoplasia [5]. To our knowledge, we present the first documented case of systemic reactive amyloidosis associated with glomus jugulare and caroticum tumors.

Serum amyloid A (SAA) is formed in the liver as an acute-phase reactant secondary to provocation of inflammation by cytokines [6]. The tumor-initiated inflammatory response prompts the hepatic production of SAA protein, resulting in AA deposition in both systemic vasculature and highly vascular organs such as the spleen, kidney, and liver [7]. Inflammatory cytokines inhibit monocyte-mediated SAA degradation in vitro, which underscores the importance of adequate SAA degradation and the influence of cytokines in secondary amyloid deposition [8]. In this case, since no other inflammatory, infectious, or neoplastic cause could be detected, we speculated that systemic reactive (AA) amyloidosis was induced by the tumor-related inflammation itself.

In patients with AA amyloidosis, if the underlying pathologic condition is controlled or eliminated, deposition of AA fibrils may be halted and perhaps even reversed [7]. This is also valid for tumor-associated AA amyloidosis based on reports [9, 10] in which resection of the AA amyloidosis-inciting tumor was followed by a marked decline in proteinuria and improvement in renal function. In the present case, since neither surgery nor radiotherapy was performed, we were not able to evaluate the response to therapy.

In conclusion, this patient had long-standing paragangliomas that resulted in eventual complications due to tumor-associated systemic amyloidosis. Paragangliomas, although rare, should be considered one of the tumors that can result in AA amyloidosis.

## 4. Summary

The patient presented here had long-standing paragangliomas that resulted in eventual complications due to tumor-associated systemic amyloidosis. Paragangliomas, although rare, should be considered one of the tumors that can result in AA amyloidosis.
